# Optimized Particle Swarm Optimization (OPSO) and its application to artificial neural network training

**DOI:** 10.1186/1471-2105-7-125

**Published:** 2006-03-10

**Authors:** Michael Meissner, Michael Schmuker, Gisbert Schneider

**Affiliations:** 1 Johann Wolfgang Goethe-Universität, Institut für Organische Chemie und Chemische Biologie, Siesmayerstraße 70, D-60323 Frankfurt, Germany

## Abstract

**Background:**

Particle Swarm Optimization (PSO) is an established method for parameter optimization. It represents a population-based adaptive optimization technique that is influenced by several "strategy parameters". Choosing reasonable parameter values for the PSO is crucial for its convergence behavior, and depends on the optimization task. We present a method for parameter meta-optimization based on PSO and its application to neural network training. The concept of the Optimized Particle Swarm Optimization (OPSO) is to optimize the free parameters of the PSO by having swarms within a swarm. We assessed the performance of the OPSO method on a set of five artificial fitness functions and compared it to the performance of two popular PSO implementations.

**Results:**

Our results indicate that PSO performance can be improved if meta-optimized parameter sets are applied. In addition, we could improve optimization speed and quality on the other PSO methods in the majority of our experiments. We applied the OPSO method to neural network training with the aim to build a quantitative model for predicting blood-brain barrier permeation of small organic molecules. On average, training time decreased by a factor of four and two in comparison to the other PSO methods, respectively. By applying the OPSO method, a prediction model showing good correlation with training-, test- and validation data was obtained.

**Conclusion:**

Optimizing the free parameters of the PSO method can result in performance gain. The OPSO approach yields parameter combinations improving overall optimization performance. Its conceptual simplicity makes implementing the method a straightforward task.

## Background

Optimizing parameters of multivariate systems is a general problem in computational biology. One of the many methods developed for parameter optimization is Particle Swarm Optimization (PSO), which was introduced by Kennedy and Eberhart in 1995 [1,2]. Emerging from simulations of dynamic systems such as bird flocks and fish swarms, the original algorithm is grounded on a stochastic search in multimodal search space. The idea of PSO is to have a swarm of particles "flying" through a multidimensional search space, looking for the global optimum. By exchanging information the particles can influence each others' movements. Each particle retains an individual (or "cognitive") memory of the best position it has visited, as well as a global (or "social") memory of the best position visited by all particles in the swarm. A particle calculates its next position based on a combination of its last movement vector, the individual and global memories, and a random component.

An advantage of PSO is its ability to handle optimization problems with multiple local optima reasonably well and its simplicity of implementation – especially in comparison to related strategies like genetic algorithms (GA). In the field of cheminformatics, PSO has successfully been applied to Quantitative Structure-Activity Relationship (QSAR) modeling, including *k*-nearest neighbor and kernel regression [3], minimum spanning tree for piecewise modeling [4], partial least squares modeling [5], and neural network training [6].

Ever since its capability to solve global optimization problems was discovered, the PSO paradigm has been developed further and improved and several variations of the original algorithm have been proposed. These include the Constriction type PSO (CPSO) [7] amongst various others (see, e.g. [6,8,9]).

The PSO algorithm itself contains some parameters which have been shown to affect its performance and convergence behavior [10-12]. Finding an optimal set of PSO parameter values is an optimization problem by itself, and thus can be dealt with by classic optimization techniques. One approach that has been pursued was based on testing various parameter combinations empirically to find one parameter set which enables PSO to handle all kinds of optimization problems reasonably well [11]. Following a different concept, Parsopoulos and Vrahatis implemented a composite PSO algorithm [13], where the Differential Evolution (DE) algorithm [14] handled the PSO heuristics online during training. They showed on a suite of test functions that their composite PSO could surpass the success rate of the plain PSO they were comparing to. They also tried to have the PSO heuristics optimized by another PSO running in parallel, but were not satisfied with preliminary results and discarded this concept in favor of the DE algorithm [13].

In this study, we present the concept of the Optimized Particle Swarm Optimization (OPSO) method. We demonstrate that it is possible to use PSO for meta-optimization of PSO heuristics. Our approach was applied to the example of artificial neural network training for the prediction of blood-brain-barrier (BBB) permeation coefficients (logBB values) of small organic molecules.

## Results and discussion

### Optimized Particle Swarm Optimization (OPSO)

The concept of the OPSO method is to have a superordinate swarm ("superswarm") optimize the parameters of subordinate swarms ("subswarms"). While the subswarms find a solution to a given optimization problem, the superswarm is used to optimize their parameters. Subswarms with parameters that are well-suited for their performance on the given optimization task will achieve a higher fitness than others. Thus, the superswarm as a whole will move to an optimal point in parameter space over time. In contrast to the approach pursued by Parsopoulos and Vrahatis [13], we used the superswarm as a wrapper for the subswarms rather than running them in parallel. As a consequence, in each epoch of the superswarm, all subswarms complete one entire optimization run on the objective function and return their fitness value to the superswarm.

**Figure 1:**
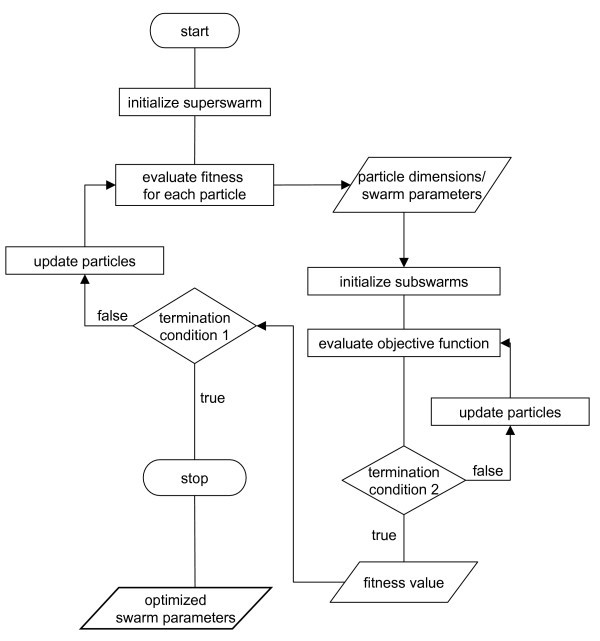
Flowchart of the OPSO method. Multiple iterations and averaging to obtain the fitness values of the subswarms are not shown. Termination conditions are problem-specific.

Our implementation of OPSO was grounded on the standard PSO implementation as defined by equation (1) (see Methods section). The dimensionality of the superswarm was determined by the number of parameters to be optimized while the subswarm's dimensionality depended on the optimization task itself. The architecture of the OPSO method is illustrated in the flowchart in Figure 1.

Sometimes subswarms perform well by chance, even if their parameters are not adapted to the problem, e.g. if the randomly initialized particles happen to be optimally placed in the search space. This results in a high fitness of an individual subswarm, although its set of parameter values may not represent an optimal point in the fitness landscape. If this happens, the superswarm will keep converging around that point unless a higher fitness of another subswarm is achieved, failing to find an optimal parameter set.

We performed multiple optimization runs per subswarm and then calculated the average of the achieved fitness values to avoid such behavior. This means, we punished parameter sets that lead to only occasional optimization success but often to failure. The more optimization runs were averaged, the more robust were the final set of optimized parameters found by OPSO. In this context the term "robust" means that the optimized set of parameters leads to an average swarm performance close to the one suggested by the OPSO optimal fitness. The optimization process went on until the superswarm met the termination condition which was a maximum number of epochs in the present study. The best solution found by the superswarm was the set of parameter values that provided the subswarms with the best performance on their optimization task.

### Optimizing PSO parameters: Experimental setup

We assessed the performance of the OPSO-method employing a suite of five different test functions (eqs. 7–11, see Methods section) and compared it to the standard PSO and the CPSO methods. The suite of test functions we used to test swarm performance consisted of five different functions, where two are unimodal (De Jong, Rosenbrock), and three are multimodal functions (Rastrigin, Schaffer F6, Griewangk). All functions except for the Schaffer function (equation 9) – which is a two-dimensional function by definition – were optimized in 30 dimensions. The task of OPSO was to find optimal swarm parameter sets for the minimization of each of the test functions. Parameters to be optimized were *w*_*start*_, *w*_*end*_, *n*_1, _and *n*_2 _(see Methods section for parameter descriptions). We initialized the dimensions of the superswarm's particles in different intervals. Subswarm parameters *w*_*start*_, *w*_*end*_, *n*_1_, *n*_2_, were initialized in the interval [0,4]. We also tried different parameterizations of the superswarm, mainly depending on the computing-time expenses of the meta-optimization. The maximum number of iterations for the subswarms was set to 1,000. A population size of 20 particles was chosen for the subswarms.

In this experiment, we decided not to use a restriction constant for the maximum velocity *V*_*max*_. As the *V*_*max *_constant is considered to be crucial for a controlled convergence behavior of standard PSO [7,15], we were interested in finding a parameter set that provided PSO with reasonable exploration and convergence capability without applying a restriction for the velocity. Therefore all remaining parameters needed to be fine-tuned to be able to provide PSO with such characteristics.

The parameters for the superswarm were chosen as defined in Table 1.

To get robust sets of parameter values, we used the average error from 15 minimization runs per test function as the fitness value. To compare the performance of OPSO with the performance of other PSO methods, we chose the standard PSO and the CPSO as reference algorithms. The configurations of those two algorithms are given in Table 2.

As no *V*_*max *_constant was used in our OPSO implementation, we also disabled it in our standard PSO implementation for this experiment. This was done to demonstrate the importance of proper calibration of parameters. However, in subsequent experiments the *V*_*max *_constant was used in order to further improve optimization quality.

For each of the five test functions and the PSO method, 400 minimization runs were performed and mean, standard deviation and median values were calculated. Thresholds as success criterion – when applied – were defined as following:

Schaffer F6, *D *= 2: mean error < 10^-5^

Griewangk, *D *= 30: mean error < 0.1

Rastrigin, *D *= 30: mean error < 100

Rosenbrock, *D *= 30: mean error < 100

Sphere, *D *= 30: mean error < 0.01

### Optimizing PSO parameters: Results

The resulting parameter sets from the meta-optimizations are given in Table 3.

For the two unimodal functions Rosenbrock and De Jong's Sphere the *n*_2_/*n*_1 _ratio was 2.14 and 2.75, respectively. These values are rather large compared to those obtained for the multimodal Griewangk (*n*_2_/*n*_1 _= 1.18), Schaffer (*n*_2_/*n*_1 _= 0.73) and Rastrigin (*n*_2_/*n*_1 _= 0.21) test functions. A large *n*_2_*/n*_1 _ratio supports faster convergence. This is because the swarm tends to concentrate on the globally best swarm position *p*_*best*, _and particles are less "distracted" by their own best positions in search space. As a consequence, the loss of diversity in the swarm population leads to a lack of global exploration. Since the unimodal functions Rosenbrock and De Jong's Sphere do not have local minima where the swarm could be trapped in, this does not have any negative side effects. On the other hand, the multimodal Griewangk, Schaffer and Rastrigin test functions have many local minima; thus a more global search is advantageous in these cases. A stronger influence of *n*_1 _supports a more diverse search and helps the swarm to avoid getting trapped in local minima.

Interestingly, the start value for the adaptive inertia weight *w*_*start *_was optimized to a negative value for the Schaffer function (Table 3). Generally, *w *being negative causes the particles to move away from the best found points in search space. Since *w*_*end *_is positive, *w *becomes positive after a certain number of iterations. Thus, the initial negative value may result in higher population diversity in the beginning of the optimization, whereas at a later stage more positive values are favored, causing a more focused exploration of the search space.

In comparison to the optimized parameters found by meta-optimization with the DE algorithm [13,14], our tuned parameters assumed different and more variable values. Parsopoulos and Vrahatis reported values for *w*, *n*_1 _and *n*_2 _that were similar to the ones proposed in earlier empirical studies [10,11,15] and had only small deviation between the different test functions they had been optimized for. Our parameter values are different from each other as indicated by high variance over different optimization runs (not shown). Remarkably, we observed that although independent meta-optimizations can produce varying parameter sets, swarm performance was not affected (not shown). A similar observation was made by Agrafiotis and coworkers [16] in QSAR feature selection, where PSO showed the capability to produce diverse solution sets of comparable quality. These authors revealed that the solution sets found by particle swarms were more variable and of higher quality at the same time compared to the ones found by Simulated Annealing. Our observation that parameter sets optimized by PSO itself seem to be more diverse than the ones obtained by Parsopoulos and Vrahatis through optimization with the DE algorithm – while providing PSO with comparable performance among each other at the same time – is in agreement with the aforementioned study.

In our view, it remains a matter of debate whether a single set of PSO coefficients can be optimal – or at least reasonable – for any kind of fitness landscapes. While it has been elegantly shown that certain coefficients can help increase the ability of a particle swarm to find optima in families of test functions [7], it seems reasonable to assume that instead of one "global" parameter set being optimal, there exist many different parameter sets leading to similar PSO performance. This speculation is substantiated by our results. Moreover, it appears intuitive to us that different fitness landscapes may require different swarm dynamics, as discussed for the example of the *n*_2_/*n*_1 _ratio above. Analysis of attractors and convergence behavior might represent a methodical approach that can lead to further clarification of this issue [7].

### Comparison with other PSO implementations

To compare OPSO with standard PSO and CPSO, 400 minimization runs were performed on our suite of test functions by each of the methods. The maximum number of epochs was fixed to 1,000. Results are summarized in Table 4.

Overall, the mean error achieved by the particle swarm with optimized parameters was smaller for four of the five functions than the one achieved by the other two PSO methods. The PSO with optimized parameters achieved a large decrease on the mean error compared to the CPSO (Schaffer: 1.6-fold; Griewangk: 6.2-fold; Rastrigin: 1.9-fold; Sphere: 56726-fold). For the minimization of the Rosenbrock function the CPSO performance was better than the OPSO performance, with an on average 1.2-fold lower final error.

The results of these statistics indicate that meta-optimization with the OPSO method does work. The parameter sets that were found by applying OPSO provided the swarms with special characteristics needed for a good optimization performance in the different fitness landscapes. Only in the case of the Rosenbrock function, the PSO with optimized parameters could not outperform the two competing methods.

Another experiment was performed in which thresholds for the mean error served as success criterions along with a maximal number of epochs. If the threshold was not reached by an optimization method within 1,000 epochs, the run was judged as failure. When it was reached, the number of epochs that were needed to reach the threshold was recorded. We employed the same swarm configurations as before, including the sets of optimized parameters. For statistical evaluation, 400 runs were performed by each PSO type, results are listed in Table 5. The PSO with optimized parameters was able to outperform the standard PSO method in all five test functions in terms of "epochs needed" and "least failures". In comparison to the constriction type PSO, the PSO with optimized parameters performed well, too. Only in two of the five test functions, OPSO did not outperform the constriction method in both "speed" and "robustness". The minimization of the Griewangk function took slightly fewer epochs with the CPSO (824) than with the OPSO (851), but had more than twice as many failures on average (CPSO: 130; OPSO: 55). On the contrary, the threshold for the Rosenbrock function was reached faster with the OPSO (195) than with the CPSO (318), but had one failure in 400 runs. The constriction method never failed to reach the threshold. For the other three test functions, the optimized PSO succeeded in reaching this criterion faster than the constriction method and having fewer failures at the same time.

### OPSO for neural network training: Experimental setup

Having demonstrated the potential usefulness of OPSO, we employed this method for training the weights and biases of two-layered neural networks. The task was to develop a quantitative prediction model for logBB values from the Lobell dataset [17]. Apart from optimizing subswarm parameters, OPSO can optimize other problem-dependent parameters simultaneously. In this part of our study, we used OPSO to optimize the number of hidden neurons *N *in the artificial neural network along with the subswarm parameters. This task has been approached by many researchers before, and various solutions to this problem have been proposed. Our aim was not to come up with a further method for network architecture optimization, but to test OPSO on a practical application.

To optimize *N*, we added another dimension to the superswarm, randomly initialized in the interval [0,50]. To obtain the actual number of hidden neurons during the meta-optimization, *N *was rounded up to the next integer.

The velocity restriction constant *V*_*max *_was also included in the meta-optimization process. The initialization interval for the *V*_*max *_dimension was [0,50]. Altogether the particles of the superswarm were six-dimensional, parameters to be optimized were *w*_*start*_, *w*_*end*_, *n*_1_, *n*_2_, *V*_*max *_and *N*. Table 6 shows the configuration of the OPSO.

For the comparison of the different PSO methods on neural network training, the configurations from Table 7 were used. We also employed the *V*_*max *_constant for our standard PSO implementation, in contrast to the previous experiment with the test functions, where we had disabled it for reasons of comparability.

### OPSO for neural network training: Results

We performed four independent OPSO runs. As network training itself showed to be more time-consuming than the minimization of the test functions, both the number of iterations and the number of particles in the superswarm were reduced in comparison to the meta-optimization of the parameters for the test functions. In addition, the fitness values for the subswarms were obtained by averaging over only three runs on the objective function, i.e. mean square error (MSE) of the neural network, instead of 15 used above in the OPSO runs on the fitness functions.

In three of the four OPSO runs, the final value for the number of hidden neurons *N *was 7, indicating a preference and possibly an optimal point in the fitness landscape. In the fourth run, *N *converged to a value of 10. The remaining parameters showed larger variance over the optimization runs, but the mean fitness values of the final solutions were comparable.

### Using optimized PSO parameters for network training on logBB data

In order to compare the performance on network training with the performance of the standard PSO and CPSO, we arbitrarily picked one out of the three parameter sets in which *N *converged to a value of 7 and used it to parameterize a PSO optimizer. The chosen parameter values are shown in Table 8. To build the quantitative prediction model, 20 two-layered nets with *N *= 7 were trained by PSO with optimized parameters. For comparison, we had PSO and CPSO train another 20 nets with identical architecture, respectively. To prevent overfitting, net weights and biases were only kept if the MSE improved on the test set.

**Figure 2:**
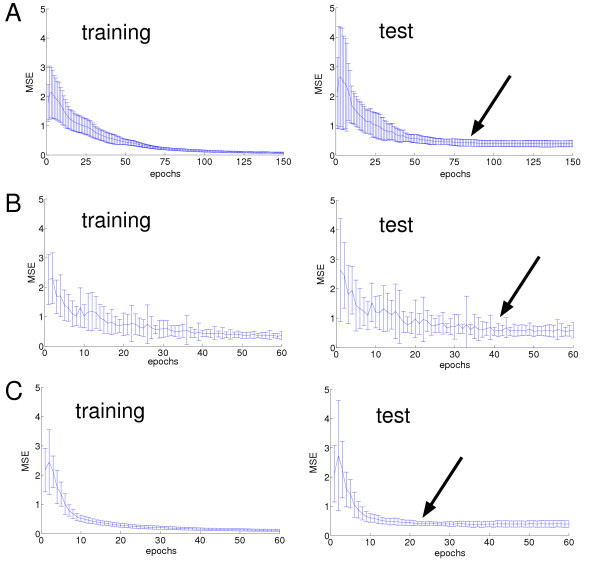
Mean MSE and standard deviation for net training of 20 neural nets with different PSO methods. A: standard PSO, B: CPSO, C: OPSO. Left: MSE for training data, right: MSE for test data. Arrows indicate approximate time of convergence.

Out of the three used PSO methods, the PSO with optimized parameters trained the nets fastest, i.e. required the smallest number of iterations. We considered the training to be finished when the MSE on the test set did not improve any further. While the standard PSO required about 80 iterations (Figure 2A) and the constriction PSO required about 40 iterations on average (Figure 2B) to finish training, the PSO with optimized parameters finished training within about 20 iterations (Figure 2C). This finding is in agreement with the data presented by Kennedy [18], who stated that it is possible to train neural nets with standard PSO within about 70 epochs, but also supposed that this could be done faster with other PSO variations.

To build a quantitative model for logBB prediction, we chose the network with the highest correlation coefficient for the test data from each of the three training sessions, respectively. For each of the three nets mean absolute error and *r*^2 ^for training and test data were calculated. The network trained by OPSO showed the highest correlation and the lowest mean absolute error for both training and test data (Table 9).

We then employed a lager dataset (courtesy of M. Nietert; manuscript in preparation; data not shown) to build a neural net for logBB prediction containing 89 structures with experimental logBB values in the training set and 44 structures with experimental logBB values in test- and validation sets, respectively. OPSO was applied, and 1,000 nets were trained with PSO using optimized parameters. The best net was selected, and its prediction capability was tested on the independent 44 validation compounds, yielding *q^2 ^*= 0.76 and a mean absolute error of 0.29. This is still in the range of the error for experimental logBB values as the mean absolute error for the experimental values is approximately 0.3 units [17].

## Conclusion

We have shown that PSO performance can be improved when its parameters are optimized specifically for the problem at hand. We have achieved this by implementing OPSO, a wrapper method for PSO, where particles of a swarm are swarms as well. An advantage of the OPSO meta-optimization method is its straightforward implementation. No other implementations than the PSO method itself are needed and only minor adaptations have to be made in order to implement the OPSO method.

In our experiments we were able to show that optimization "speed" as well as "robustness" can be improved by applying optimized parameters to PSO. Similar observations were made when deploying OPSO to a real life application such as artificial neural network training. Our results indicate that fast and efficient net training is possible with optimized parameters. Moreover, through parameter optimization training time can be decreased while training success may be increased at the same time. Another feature of OPSO is that other problem-dependent, non-PSO parameters can be optimized along with the PSO parameters. We have tested this on the example of the number of hidden neurons in a two-layered neural net. While it has not been verified that the chosen number of seven hidden neurons was optimal, three out of four OPSO runs resulted in that same number which indicates that seven neurons might be a preferred network configuration for the particular task.

We have shown for one sample implementation of PSO that the basic OPSO architecture actually works. Although there seem to be more powerful implementations of PSO (such as CPSO) than the standard implementation which we used, it was still possible to outperform the constriction type simply by using optimized parameters in a standard PSO. Since the OPSO method is not limited on the use of standard PSO, it should be possible to implement it with any PSO algorithm with the aim to improve their performance. For example, instead of a global PSO version a local version could be used and the size of the neighborhood could be included in the meta-optimization process.

Typical areas of application include optimization of a large number of problems with similar fitness landscapes. The OPSO would be run on some exemplary problem instances, and the resulting optimized swarm parameters could be used to treat the remaining instances. For example, in order to model quantitative structure-activity relationships from a large compound database as obtained from high-throughput screening, one could first select a small representative subset of compounds and have OPSO train a neural network. The resulting optimized swarm parameters can then be used for network training on the entire database. Our results suggest that not only network training would converge faster, but might also lead to more robust results in cross-validation.

## Methods

### Particle swarm optimization (PSO)

Each particle was initialized at a random position in search space. The position of particle *i *is given by the vector *x*_*i *_= (*x*_*i*1_,*x*_*i*2_, ..., *x*_*iD*_) where *D *is the dimensionality of the problem. Its velocity is given by the vector *v*_*i *_= (*v*_*i*1_, *v*_*i*2_, ..., *v*_*iD*_).

Two kinds of memory were implemented that influence the movement of the particles: In the cognitive memory *p*_*i *_*= (p*_*i*1_*, p*_*i*2_, ..., *p*_*iD*_*) *the best previous position visited by each individual particle *i *is stored. The vector *p*_*best*_*= (p*_*best*1_*, p*_*best*2_, ..., *p*_*bestD*_*)*, also called "social memory", contains the position of the best point in search space visited by all swarm particles so far.

In each epoch the particle velocities were updated according to equation (1):

v_*i*_(*t*+1) = *w· *v_*i*_(*t*)+ *n*_1· _*r*_1·_(*p*_*i*_-*x*_*i*_(*t*))+*n*_2·_*r*_2· _(*p*_*best *_- *x*_*i*_(*t*)),     (1)

where *w *is the inertia weight, a weighting factor for the velocity, *n*_1 _and *n*_2 _are positive constants called "cognitive" and "social" parameter weighting the influence of the two different swarm memories, and *r*_1 _and *r*_2 _are random numbers between 0 and 1.

In some of our experiments a restriction constant *V*_*max *_was applied to control the velocity of particles (cf. Results section). Velocities exceeding the threshold set by *V*_*max *_were set back to the threshold value.

The inertia weight *w *can either be implemented as constant or in a way so that its value is changed linearly with time. A start and end value is set and for each epoch a new value of *w *is calculated as in equation (2).

w=wstart−wstart−wendMaxEpochs⋅Epochs,     (2)
 MathType@MTEF@5@5@+=feaafiart1ev1aaatCvAUfKttLearuWrP9MDH5MBPbIqV92AaeXatLxBI9gBaebbnrfifHhDYfgasaacH8akY=wiFfYdH8Gipec8Eeeu0xXdbba9frFj0=OqFfea0dXdd9vqai=hGuQ8kuc9pgc9s8qqaq=dirpe0xb9q8qiLsFr0=vr0=vr0dc8meaabaqaciaacaGaaeqabaqabeGadaaakeaacqWG3bWDcqGH9aqpcqWG3bWDdaWgaaWcbaGaem4CamNaemiDaqNaemyyaeMaemOCaiNaemiDaqhabeaakiabgkHiTmaalaaabaGaem4DaC3aaSbaaSqaaiabdohaZjabdsha0jabdggaHjabdkhaYjabdsha0bqabaGccqGHsislcqWG3bWDdaWgaaWcbaGaemyzauMaemOBa4MaemizaqgabeaaaOqaaiabd2eanjabdggaHjabdIha4jabdweafjabdchaWjabd+gaVjabdogaJjabdIgaOjabdohaZbaacqGHflY1cqWGfbqrcqWGWbaCcqWGVbWBcqWGJbWycqWGObaAcqWGZbWCcqGGSaalcaWLjaGaaCzcaiabcIcaOiabikdaYiabcMcaPaaa@6322@

where *w*_*start *_is the initial value for *w *and *w*_*end *_is the terminal value. *Epochs *stand for the actual number of epochs and *MaxEpochs *is the maximum number of epochs for the optimization.

The advantage of an adaptive inertia weight is that swarm behavior can be varied and adapted over time. Often a bigger start value than end value is applied, causing the swarm to perform a more global search with large movements in the beginning and shifting to smaller movements and fine tuning in the end of the optimization process.

After the velocity vector had been calculated the positions of the particles were updated according to equation (3)

*x*_*i*_(*t*+1) = *x*_*i*_(*t*)+*v*_*i*_(*t*+1).     (3)

We employed a maximum number of epochs as termination condition for the algorithm, depending on the task also in combination with a threshold as success criterion.

### Constriction type PSO

Another common implementation of PSO is the constriction type PSO [7]. In the following, we refer to this PSO variant as "CPSO". The velocity vector was calculated according to equation (4):

v_*i*_(*t*+1) = *K· *(v_*i*_(*t*)+*n*_1· _*r*_2·_(*p*_*i *_- *x*_*i*_(*t*)) + *n*_2·_*r*_2· _(*p*_*best *_- *x*_*i*_(*t*))),     (4)

with the constriction factor *K *as defined in equation (5):

K=2|2−ϕ−ϕ2−4ϕ|,     (5)
 MathType@MTEF@5@5@+=feaafiart1ev1aaatCvAUfKttLearuWrP9MDH5MBPbIqV92AaeXatLxBI9gBaebbnrfifHhDYfgasaacH8akY=wiFfYdH8Gipec8Eeeu0xXdbba9frFj0=OqFfea0dXdd9vqai=hGuQ8kuc9pgc9s8qqaq=dirpe0xb9q8qiLsFr0=vr0=vr0dc8meaabaqaciaacaGaaeqabaqabeGadaaakeaacqWGlbWscqGH9aqpdaWcaaqaaiabikdaYaqaaiabcYha8jabikdaYiabgkHiTiabew9aQjabgkHiTmaakaaabaGaeqy1dO2aaWbaaSqabeaacqaIYaGmaaGccqGHsislcqaI0aancqaHvpGAaSqabaGccqGG8baFaaGaeiilaWIaaCzcaiaaxMaacqGGOaakcqaI1aqncqGGPaqkaaa@4302@

and ϕ is computed as follows (equation (6)):

ϕ = *n*_1 _+ *n*_2_, ϕ >4.     (6)

The constriction factor *K *controls the magnitude of the particle velocity and can be seen as a dampening factor. It provides the algorithm with two important features [15]: First, it usually leads to a faster convergence than standard PSO. Second, the swarm keeps the ability to perform wide movements in search space even if convergence is already advanced but a new optimum is found. Therefore the CPSO has a potential ability to avoid being trapped into local optima while possessing a fast convergence capability and was shown to have superior performance than the standard PSO [15].

### Test functions

*Rastrigin*:

f(x)=10⋅D+∑i=1D(xi2−10⋅cos⁡(2⋅π⋅xi)),     (7)
 MathType@MTEF@5@5@+=feaafiart1ev1aaatCvAUfKttLearuWrP9MDH5MBPbIqV92AaeXatLxBI9gBaebbnrfifHhDYfgasaacH8akY=wiFfYdH8Gipec8Eeeu0xXdbba9frFj0=OqFfea0dXdd9vqai=hGuQ8kuc9pgc9s8qqaq=dirpe0xb9q8qiLsFr0=vr0=vr0dc8meaabaqaciaacaGaaeqabaqabeGadaaakeaacqWGMbGzcqGGOaakcqWG4baEcqGGPaqkcqGH9aqpcqaIXaqmcqaIWaamcqGHflY1cqWGebarcqGHRaWkdaaeWbqaamaabmaabaGaemiEaG3aa0baaSqaaiabdMgaPbqaaiabikdaYaaakiabgkHiTiabigdaXiabicdaWiabgwSixlGbcogaJjabc+gaVjabcohaZjabcIcaOiabikdaYiabgwSixlabec8aWjabgwSixJqaciab=Hha4naaBaaaleaacqWFPbqAaeqaaOGaeiykaKcacaGLOaGaayzkaaaaleaacqWGPbqAcqGH9aqpcqaIXaqmaeaacqWGebara0GaeyyeIuoakiabcYcaSiaaxMaacaWLjaGaeiikaGIaeG4naCJaeiykaKcaaa@5E8E@

global minimum: *f*(*x*) = 0, *x*_*i *_= 0.

De Jong's Sphere:

f(x)=∑i=1Dxi2,     (8)
 MathType@MTEF@5@5@+=feaafiart1ev1aaatCvAUfKttLearuWrP9MDH5MBPbIqV92AaeXatLxBI9gBaebbnrfifHhDYfgasaacH8akY=wiFfYdH8Gipec8Eeeu0xXdbba9frFj0=OqFfea0dXdd9vqai=hGuQ8kuc9pgc9s8qqaq=dirpe0xb9q8qiLsFr0=vr0=vr0dc8meaabaqaciaacaGaaeqabaqabeGadaaakeaacqWGMbGzcqGGOaakcqWG4baEcqGGPaqkcqGH9aqpdaaeWbqaaiabdIha4naaDaaaleaacqWGPbqAaeaacqaIYaGmaaGccqGGSaalcaWLjaGaaCzcaiabcIcaOiabiIda4iabcMcaPaWcbaGaemyAaKMaeyypa0JaeGymaedabaGaemiraqeaniabggHiLdaaaa@41A7@

global minimum: *f*(*x*) = 0, *x*_*i *_= 0.

Schaffer F6:

f(x)=0.5+sin⁡2x2+y2−0.5(1+0.001⋅(x2+y2))2,     (9)
 MathType@MTEF@5@5@+=feaafiart1ev1aaatCvAUfKttLearuWrP9MDH5MBPbIqV92AaeXatLxBI9gBaebbnrfifHhDYfgasaacH8akY=wiFfYdH8Gipec8Eeeu0xXdbba9frFj0=OqFfea0dXdd9vqai=hGuQ8kuc9pgc9s8qqaq=dirpe0xb9q8qiLsFr0=vr0=vr0dc8meaabaqaciaacaGaaeqabaqabeGadaaakeaacqWGMbGzcqGGOaakcqWG4baEcqGGPaqkcqGH9aqpcqaIWaamcqGGUaGlcqaI1aqncqGHRaWkdaWcaaqaaiGbcohaZjabcMgaPjabc6gaUnaaCaaaleqabaGaeGOmaidaaOWaaOaaaeaacqWG4baEdaahaaWcbeqaaiabikdaYaaakiabgUcaRiabdMha5naaCaaaleqabaGaeGOmaidaaaqabaGccqGHsislcqaIWaamcqGGUaGlcqaI1aqnaeaadaqadaqaaiabigdaXiabgUcaRiabicdaWiabc6caUiabicdaWiabicdaWiabigdaXiabgwSixpaabmaabaGaemiEaG3aaWbaaSqabeaacqaIYaGmaaGccqGHRaWkcqWG5bqEdaahaaWcbeqaaiabikdaYaaaaOGaayjkaiaawMcaaaGaayjkaiaawMcaamaaCaaaleqabaGaeGOmaidaaaaakiabcYcaSiaaxMaacaWLjaGaeiikaGIaeGyoaKJaeiykaKcaaa@5D27@

global minimum: *f*(*x*) = 0, *x*_*i *_= 0.

Rosenbrock:

f(x)=∑i=1D−1100⋅(xi+1−xi2)2+(1−xi)2,     (10)
 MathType@MTEF@5@5@+=feaafiart1ev1aaatCvAUfKttLearuWrP9MDH5MBPbIqV92AaeXatLxBI9gBaebbnrfifHhDYfgasaacH8akY=wiFfYdH8Gipec8Eeeu0xXdbba9frFj0=OqFfea0dXdd9vqai=hGuQ8kuc9pgc9s8qqaq=dirpe0xb9q8qiLsFr0=vr0=vr0dc8meaabaqaciaacaGaaeqabaqabeGadaaakeaacqWGMbGzcqGGOaakcqWG4baEcqGGPaqkcqGH9aqpdaaeWbqaaiabigdaXiabicdaWiabicdaWiabgwSixdWcbaGaemyAaKMaeyypa0JaeGymaedabaGaemiraqKaeyOeI0IaeGymaedaniabggHiLdGcdaqadaqaaiabdIha4naaBaaaleaacqWGPbqAcqGHRaWkcqaIXaqmaeqaaOGaeyOeI0IaemiEaG3aa0baaSqaaiabdMgaPbqaaiabikdaYaaaaOGaayjkaiaawMcaamaaCaaaleqabaGaeGOmaidaaOGaey4kaSYaaeWaaeaacqaIXaqmcqGHsislcqWG4baEdaWgaaWcbaGaemyAaKgabeaaaOGaayjkaiaawMcaamaaCaaaleqabaGaeGOmaidaaOGaeiilaWIaaCzcaiaaxMaacqGGOaakcqaIXaqmcqaIWaamcqGGPaqkaaa@5A7A@

global minimum:*f*(*x*) = 0, *x*_*i *_= 1.

Griewangk:

f(x)=∑i=1Dxi24000−∏i=1Dcos⁡(xii)+1,     (11)
 MathType@MTEF@5@5@+=feaafiart1ev1aaatCvAUfKttLearuWrP9MDH5MBPbIqV92AaeXatLxBI9gBaebbnrfifHhDYfgasaacH8akY=wiFfYdH8Gipec8Eeeu0xXdbba9frFj0=OqFfea0dXdd9vqai=hGuQ8kuc9pgc9s8qqaq=dirpe0xb9q8qiLsFr0=vr0=vr0dc8meaabaqaciaacaGaaeqabaqabeGadaaakeaacqWGMbGzcqGGOaakcqWG4baEcqGGPaqkcqGH9aqpdaaeWbqaamaalaaabaGaemiEaG3aa0baaSqaaiabdMgaPbqaaiabikdaYaaaaOqaaiabisda0iabicdaWiabicdaWiabicdaWaaacqGHsisldaqeWbqaaiGbcogaJjabc+gaVjabcohaZnaabmaabaWaaSaaaeaacqWG4baEdaWgaaWcbaGaemyAaKgabeaaaOqaamaakaaabaGaemyAaKgaleqaaaaaaOGaayjkaiaawMcaaiabgUcaRiabigdaXiabcYcaSiaaxMaacaWLjaGaeiikaGIaeGymaeJaeGymaeJaeiykaKcaleaacqWGPbqAcqGH9aqpcqaIXaqmaeaacqWGebara0Gaey4dIunaaSqaaiabdMgaPjabg2da9iabigdaXaqaaiabdseaebqdcqGHris5aaaa@59F2@

global minimum:*f*(*x*) = 0, *x*_*i *_= 0.

*D *denotes the number of dimensions. With the exception of the two-dimensional Schaffer F6 function, all other functions were used in 30 dimensions for the minimizations.

Initialization intervals were chosen as follows:

Rastrigin: [5.12,-5.12]

Sphere: [100,-100]

Schaffer F6: [100,-100]

Rosenbrock: [2.048,-2.048]

Griewangk: [600,-600]

The mean error for the minimization of the test functions was calculated as in equation (12):

mean error=1j⋅∑1j|f(xj)−f(xopt)|,     (12)
 MathType@MTEF@5@5@+=feaafiart1ev1aaatCvAUfKttLearuWrP9MDH5MBPbIqV92AaeXatLxBI9gBaebbnrfifHhDYfgasaacH8akY=wiFfYdH8Gipec8Eeeu0xXdbba9frFj0=OqFfea0dXdd9vqai=hGuQ8kuc9pgc9s8qqaq=dirpe0xb9q8qiLsFr0=vr0=vr0dc8meaabaqaciaacaGaaeqabaqabeGadaaakeaacqWGTbqBcqWGLbqzcqWGHbqycqWGUbGBcaaMc8UaemyzauMaemOCaiNaemOCaiNaem4Ba8MaemOCaiNaeyypa0ZaaSaaaeaacqaIXaqmaeaacqWGQbGAaaGaeyyXIC9aaabCaeaadaabdaqaaiabdAgaMjabcIcaOiabdIha4naaBaaaleaacqWGQbGAaeqaaOGaeiykaKIaeyOeI0IaemOzayMaeiikaGIaemiEaG3aaSbaaSqaaiabd+gaVjabdchaWjabdsha0bqabaGccqGGPaqkaiaawEa7caGLiWoacqGGSaalcaWLjaGaaCzcaiabcIcaOiabigdaXiabikdaYiabcMcaPaWcbaGaeGymaedabaGaemOAaOganiabggHiLdaaaa@5DAB@

where *j *is the number of runs performed, *f(x*_*j*_*) *is the function value for each solution found in minimization run *j *and *f(x*_*opt*_*) *is the function value at the global minimum.

### Multilayer artificial neural networks

We used two-layered, feed-forward artificial neural networks (ANN) to predict logBB values. Such networks represent universal function approximators [19] and have been described in detail elsewhere [20]. Briefly, a network with *k *inputs, *j *neurons in the hidden layer and *i *output neurons delivers the output Oiμ
 MathType@MTEF@5@5@+=feaafiart1ev1aaatCvAUfKttLearuWrP9MDH5MBPbIqV92AaeXatLxBI9gBaebbnrfifHhDYfgasaacH8akY=wiFfYdH8Gipec8Eeeu0xXdbba9frFj0=OqFfea0dXdd9vqai=hGuQ8kuc9pgc9s8qqaq=dirpe0xb9q8qiLsFr0=vr0=vr0dc8meaabaqaciaacaGaaeqabaqabeGadaaakeaacqWGpbWtdaqhaaWcbaGaemyAaKgabaGaeqiVd0gaaaaa@3111@ in response to a pattern *μ *according to equation (13).

Oiμ=go(bi+∑jWijgH(bj+∑kwjkξkμ)),     (13)
 MathType@MTEF@5@5@+=feaafiart1ev1aaatCvAUfKttLearuWrP9MDH5MBPbIqV92AaeXatLxBI9gBaebbnrfifHhDYfgasaacH8akY=wiFfYdH8Gipec8Eeeu0xXdbba9frFj0=OqFfea0dXdd9vqai=hGuQ8kuc9pgc9s8qqaq=dirpe0xb9q8qiLsFr0=vr0=vr0dc8meaabaqaciaacaGaaeqabaqabeGadaaakeaacqWGpbWtdaqhaaWcbaGaemyAaKgabaGaeqiVd0gaaOGaeyypa0Jaem4zaC2aaSbaaSqaaiabd+gaVbqabaGcdaqadaqaaiabdkgaInaaBaaaleaacqWGPbqAaeqaaOGaey4kaSYaaabuaeaacqWGxbWvdaWgaaWcbaGaemyAaKMaemOAaOgabeaakiabdEgaNnaaBaaaleaacqWGibasaeqaaOWaaeWaaeaacqWGIbGydaWgaaWcbaGaemOAaOgabeaakiabgUcaRmaaqafabaGaem4DaC3aaSbaaSqaaiabdQgaQjabdUgaRbqabaGccqaH+oaEdaqhaaWcbaGaem4AaSgabaGaeqiVd0gaaaqaaiabdUgaRbqab0GaeyyeIuoaaOGaayjkaiaawMcaaaWcbaGaemOAaOgabeqdcqGHris5aaGccaGLOaGaayzkaaGaeiilaWIaaCzcaiaaxMaacqGGOaakcqaIXaqmcqaIZaWmcqGGPaqkaaa@5CA7@

with g_*o*_, g_*H *_the transfer functions of the output and hidden layer neurons (*vide infra*), *b*_*i*_, *b*_*j *_the bias of the neurons, *W*_*ij*_the weight of the *j*th hidden neuron to the *i*th output neuron, *w*_*jk *_the weight of *k*th input neuron to the *j*th hidden neuron, and ξkμ
 MathType@MTEF@5@5@+=feaafiart1ev1aaatCvAUfKttLearuWrP9MDH5MBPbIqV92AaeXatLxBI9gBaebbnrfifHhDYfgasaacH8akY=wiFfYdH8Gipec8Eeeu0xXdbba9frFj0=OqFfea0dXdd9vqai=hGuQ8kuc9pgc9s8qqaq=dirpe0xb9q8qiLsFr0=vr0=vr0dc8meaabaqaciaacaGaaeqabaqabeGadaaakeaacqaH+oaEdaqhaaWcbaGaem4AaSgabaGaeqiVd0gaaaaa@31B1@ the *k*th element of input pattern *μ*. In the hidden layer, we used a sigmoidal transfer function (equation (14))

gH(x)=11+e−x,     (14)
 MathType@MTEF@5@5@+=feaafiart1ev1aaatCvAUfKttLearuWrP9MDH5MBPbIqV92AaeXatLxBI9gBaebbnrfifHhDYfgasaacH8akY=wiFfYdH8Gipec8Eeeu0xXdbba9frFj0=OqFfea0dXdd9vqai=hGuQ8kuc9pgc9s8qqaq=dirpe0xb9q8qiLsFr0=vr0=vr0dc8meaabaqaciaacaGaaeqabaqabeGadaaakeaacqWGNbWzdaWgaaWcbaGaemisaGeabeaakiabcIcaOiabdIha4jabcMcaPiabg2da9maalaaabaGaeGymaedabaGaeGymaeJaey4kaSIaemyzau2aaWbaaSqabeaacqGHsislcqWG4baEaaaaaOGaeiilaWIaaCzcaiaaxMaacqGGOaakcqaIXaqmcqaI0aancqGGPaqkaaa@4001@

where *x *is the net input of a neuron.

For the output neuron the linear transfer function from equation (15) was used:

g_*o*_(*x*) = *x*.     (15)

During training, network performance was assessed using the mean square error (MSE) computed as the squared difference between the predicted values *O*_predict _and the expected values (target values) *O*_expect _(equation (16)) for a number of predictions *S*. In this study, target values were experimentally determined logBB values ([17]).

MSE(Oexpect,Opredict)=1S∑i=1S(Oexpect−Opredict)2.     (16)
 MathType@MTEF@5@5@+=feaafiart1ev1aaatCvAUfKttLearuWrP9MDH5MBPbIqV92AaeXatLxBI9gBaebbnrfifHhDYfgasaacH8akY=wiFfYdH8Gipec8Eeeu0xXdbba9frFj0=OqFfea0dXdd9vqai=hGuQ8kuc9pgc9s8qqaq=dirpe0xb9q8qiLsFr0=vr0=vr0dc8meaabaqaciaacaGaaeqabaqabeGadaaakeaacqWGnbqtcqWGtbWucqWGfbqrcqGGOaakcqWGpbWtdaWgaaWcbaacbaGae8xzauMae8hEaGNae8hCaaNae8xzauMae83yamMae8hDaqhabeaakiabcYcaSiabd+eapnaaBaaaleaacqWFWbaCcqWFYbGCcqWFLbqzcqWFKbazcqWFPbqAcqWFJbWycqWF0baDaeqaaOGaeiykaKIaeyypa0ZaaSaaaeaacqaIXaqmaeaacqWGtbWuaaWaaabCaeaadaqadaqaaiabd+eapnaaBaaaleaacqWFLbqzcqWF4baEcqWFWbaCcqWFLbqzcqWFJbWycqWF0baDaeqaaOGaeyOeI0Iaem4ta80aaSbaaSqaaiab=bhaWjab=jhaYjab=vgaLjab=rgaKjab=LgaPjab=ngaJjab=rha0bqabaaakiaawIcacaGLPaaadaahaaWcbeqaaiabikdaYaaakiabc6caUiaaxMaacaWLjaGaeiikaGIaeGymaeJaeGOnayJaeiykaKcaleaacqWGPbqAcqGH9aqpcqaIXaqmaeaacqWGtbWua0GaeyyeIuoaaaa@6ECC@

The quality of quantitative predictions of logBB values was estimated using Pearson's correlation coefficient *r *(equation (17)).

r(i,j)=C(i,j)C(i,i)⋅C(j,j),     (17)
 MathType@MTEF@5@5@+=feaafiart1ev1aaatCvAUfKttLearuWrP9MDH5MBPbIqV92AaeXatLxBI9gBaebbnrfifHhDYfgasaacH8akY=wiFfYdH8Gipec8Eeeu0xXdbba9frFj0=OqFfea0dXdd9vqai=hGuQ8kuc9pgc9s8qqaq=dirpe0xb9q8qiLsFr0=vr0=vr0dc8meaabaqaciaacaGaaeqabaqabeGadaaakeaacqWGYbGCcqGGOaakcqWGPbqAcqGGSaalcqWGQbGAcqGGPaqkcqGH9aqpdaWcaaqaaiabdoeadjabcIcaOiabdMgaPjabcYcaSiabdQgaQjabcMcaPaqaamaakaaabaGaem4qamKaeiikaGIaemyAaKMaeiilaWIaemyAaKMaeiykaKIaeyyXICTaem4qamKaeiikaGIaemOAaOMaeiilaWIaemOAaOMaeiykaKcaleqaaaaakiabcYcaSiaaxMaacaWLjaGaeiikaGIaeGymaeJaeG4naCJaeiykaKcaaa@4FB5@

with C(*i, j*) the covariance matrix of two vectors *i *and *j*.

During training, the network weights *W *and biases *b *were adapted using different swarm algorithms with MSE as performance function. We kept record of the network parameters in every training epoch (*vide supra*). In order to insure generalization ability, the final network parameters were taken from the epoch before the performance on the test data started to degrade. We used the MATLAB Neural Network Toolbox for all ANN-related tasks [21].

### Data sets

For a test of OPSO performance on a real world problem, we trained ANNs on the Lobell data set [17], containing 65 molecules with experimental logBB values. The data was divided into test and training set as described [17]. This resulted in 48 molecules in the training set and 17 molecules in the test set. In a pre-processing step, hydrogens were removed with the CLIFF software [22] and for each molecule the 150 standard CATS topological pharmacophore descriptors [23] were calculated with the *speedCATS *software [24]. All descriptors with a standard deviation of zero were removed, resulting in 98 descriptors that were used as inputs for the neural networks.

### Web based Java Applet

A Java Applet termed "PsoVis" for the three-dimensional visualization of particle swarm optimization implementing PSO and CPSO is available on our gecco^® ^server the world-wide-web [25].

## List of abbreviations

ANN Artificial neural network

BBB Blood-brain barrier

CPSO Constriction-type particle swarm optimization

D Dimensions

DE Differential Evolution

GA Genetic algorithm

K Constriction factor

logBB Logarithm of the blood-brain barrier permeation coefficient

MSE Mean square error

PSO Particle swarm optimization

OPSO Optimized particle swarm optimization

QSAR Quantitative structure-activity relationship

## Authors' contributions

M. Meissner implemented the particle swarm algorithms and the Java applet and performed the experiments. M. Schmuker participated in algorithm design and application. G. Schneider conceived of the study, and participated in its design and coordination. All authors contributed to manuscript preparation, and read and approved the final manuscript.

## Figures and Tables

**Table 1:** Swarm configurations of super- and subswarms.

**Swarm parameters**	**Superswarm**	**Subswarms**
max. number of iterations	100	1000
Number of particles	30	20
*W*	0.5	-
*N*_1_	2	-
*N*_2_	2	-
*V*_*max*_	20	-

**Table 2:** Swarm configurations of the compared PSO methods.

**Swarm parameters**	**Standard type PSO**	**Constriction-type PSO**	**PSO with OPSO-parameters**
max. number of iterations	1000	1000	1000
number of particles	20	20	20
*w*_*start*_	0.9	-	optimized
*w*_*end*_	0.4	-	optimized
*N*_1_	2	2.05	optimized
*N*_2_	2	2.05	optimized
constriction factor *k*	-	0.73	-

**Table 3:** Optimized swarm parameters for the five test functions.

**Optimized parameters**	**Schaffer D = 2**	**Griewangk D = 30**	**Rastrigin D = 30**	**Rosenbrock D = 30**	**Sphere D = 30**
*w*_*start*_	-0.19	0.68	0.76	0.08	0.147
*w*_*end*_	1.57	0.18	0.85	0.63	0.070
*n*_1_	0.66	1.87	1.89	1.20	0.984
*n*_2_	0.48	2.21	0.40	2.57	2.71

**Table 4:** Mean error, standard deviation and median error of a standard type PSO, CPSO, and OPSO implementation. Particle swarms with 20 particles, 1,000 epochs. Best performance (i.e., lowest error) for each function is highlighted in bold letters.

	**Schaffer F6 D = 2**	**Griewangk D = 30**	**Rastrigin D = 30**	**Rosenbrock D = 30**	**Sphere D = 30**
**standard type PSO**					
mean error	0.0042	0.827	99.5	91.5	4.14
standard deviation	0.0048	0.361	27.0	47.2	6.56
median error	**0**	0.914	98.2	85.1	1.89
**CPSO**					
mean error	0.0048	0.148	86.2	**32.2**	0.0035
standard deviation	0.0049	0.616	23.0	19.8	0.070
median error	3.89·10^-7^	0.039	84.6	**24.6**	3.2·10^-8^
**PSO with optimized parameters**					
mean error	**0.0030**	**0.024**	**46.5**	37.4	**6.17·10**^-8^
standard deviation	0.0045	0.040	13.1	24.2	5.53·10^-7^
Median error	1.91·10^-8^	**0.015**	**44.8**	25.8	**1.02·10**^-9^

**Table 5:** Mean number of epochs until the minimization threshold was reached and mean number of failures.

	**Schaffer F6 *D *= 2**	**Griewangk *D *= 30**	**Rastrigin *D *= 30**	**Rosenbrock *D *= 30**	**Sphere *D *= 30**
**standard type PSO**					
mean number of epochs	808	1000	958	955	1000
number of failures	183	400	179	147	400
**CPSO**					
mean number of epochs	727	**824**	507	318	485
number of failures	255	130	119	**0**	1
**OPSO**					
mean number of epochs	**715**	851	**194**	**195**	**306**
number of failures	**147**	**55**	**0**	1	**0**

**Table 6:** Swarm configurations of super- and subswarms for neural network training.

**Swarm parameters**	**Superswarm**	**Subswarms**
max. number of iterations	20	60
Number of particles	10	20
*w*_*start*_	0.9	optimized
*w*_*end*_	0.4	optimized
*n*_1_	1.3	optimized
*n*_2_	1.7	optimized
*V*_*max*_	-	optimized

**Table 7:** Swarm configurations of the compared PSO methods.

**Swarm parameters**	**Standard type PSO**	**CPSO**	**PSO with OPSO-parameters**
max. number of iterations	150	60	60
Number of particles	20	20	20
*w*_*start*_	0.9	-	optimized
*w*_*end*_	0.4	-	optimized
*n*_1_	2	2.05	optimized
*n*_2_	2	2.05	optimized
*V*_*max*_	20	-	optimized
constriction factor *k*	-	0.73	-

**Table 8:** Optimized parameter values for neural network training.* N *is the number of hidden neurons.

**Parameters**	**optimized values**
*w*_*start*_	2.95
*w*_*end*_	-0.1
*n*_1_	2.82
*n*_2_	12.5
*V*_*max*_	13.2
*N*	7

**Table 9:** Comparison of different quantitative models for logBB prediction.

	**Best OPSO net**	**Best standard type PSO net**	**Best CPSO net**
**Training data**			
mean absolute error	0.23	0.31	0.39
*R^2^*	0.87	0.76	0.62
**Test data**			
mean absolute error	0.25	0.3	0.39
*R^2^*	0.87	0.73	0.59

## References

[B1] Kennedy J, Eberhart RC (1995). Particle swarm optimization. Proceedings of IEEE International Conference on Neural Networks; Piscataway, NJ.

[B2] Eberhart RC, Kennedy J (1995). A new optimizer using particle swarm theory. Proceedings of the Sixth International Symposium on Micromachine and Human Science.

[B3] Cedeno W, Agrafiotis DK (2003). Using particle swarms for the development of QSAR models based on K-nearest neighbor and kernel regression. J Comput Aided Mol Des.

[B4] Shen Q, Jiang JH, Jiao CX, Huan SY, Shen GL, Yu RQ (2004). Optimized partition of minimum spanning tree for piecewise modeling by particle swarm algorithm. QSAR studies of antagonism of angiotensin II antagonists. J Chem Inf Comput Sci.

[B5] Lin W, Jiang J, Shen Q, Shen G, Yu R (2005). Optimized block-wise variable combination by particle swarm optimization for partial least squares modeling in quantitative structure-activity relationship studies. J Chem Inf Model.

[B6] Shen Q, Jiang JH, Jiao CX, Lin WQ, Shen GL, Yu RQ (2004). Hybridized particle swarm algorithm for adaptive structure training of multilayer feed-forward neural network: QSAR studies of bioactivity of organic compounds. J Comput Chem.

[B7] Clerc M, Kennedy J (2002). The Particle Swarm – Explosion, Stability, and Convergence in a Multidimensional Complex Space. IEEE Transactions on Evolutionary Computation;.

[B8] Rasmussen TK, Krink T (2003). Improved Hidden Markov Model training for multiple sequence alignment by a particle swarm optimization-evolutionary algorithm hybrid. Biosystems.

[B9] Veeramachaneni K, Peram T, Mohan CK, Osadciw LA (2003). Optimization Using Particle Swarms with Near Neighbor Interactions. Lecture Notes in Computer Science (LNCS) No 2723: Proceedings of the Genetic and Evolutionary Computation Conference (GECCO).

[B10] Shi Y, Eberhart RC (1998). Parameter selection in particle swarm optimization. Evolutionary Programming VII: Proceedings of the Seventh Annual Conference on Evolutionary Programming.

[B11] Carlisle A, Dozier G (2001). An Off-The-Shelf PSO. Proceedings of the Workshop on Particle Swarm Optimization 2001; Indianapolis, IN.

[B12] Bergh vdF, Engelbrecht AP (2001). Effects of Swarm Size on Cooperative Particle Swarm Optimizers. Proceedings of the Genetic and Evolutionary Computation Conference (GECCO).

[B13] Parsopoulos KE, Vrahatis MN (2002). Recent approaches to global optimization problems through Particle Swarm Optimization. Natural Computing.

[B14] Storn R, Price K (1997). Differential evolution – a simple and efficient heuristic for global optimization over continuous spaces. J Global Optimization.

[B15] Eberhart RC, Shi Y (2000). Comparing inertia weights and constriction factors in Particle Swarm Optimization. Proceedings of the Congress on Evolutionary Computating.

[B16] Agrafiotis DK, Cedeno W (2002). Feature Selection for Structure-Activity Correlation Using Binary Particle Swarms. J Med Chem.

[B17] Lobell M, Molnar L, Keseru GM (2003). Recent advances in the prediction of blood-brain partitioning from molecular structure. J Pharm Sci.

[B18] Kennedy J, Eberhart RC (2001). Swarm Intelligence.

[B19] Werbos P (1974). Beyond Regression: New Tools for Prediction and Analysis in the Behavioral Sciences.

[B20] Hertz J, Palmer RG, Krogh AS (1991). Introduction to the theory of neural computation.

[B21] Matlab. Version 650, The MathWorks, Inc.

[B22] CLIFF Version 114.

[B23] Schneider G, Neidhart W, Giller T, Schmid G (1999). Scaffold-Hopping by Topological Pharmacophore Search: A Contribution to Virtual Screening. Angew Chem Int Ed Engl.

[B24] Fechner U, Franke L, Renner S, Schneider P, Schneider G (2003). Comparison of correlation vector methods for ligand-based similarity searching. J Comput Aided Mol Des.

[B25] (2003). PsoVis. http://gecco.org.chemie.uni-frankfurt.de/PsoVis/index.html.

